# Small-angle X-ray scattering analysis of poly(amic acid) dispersed in a liquid matrix to understand the size control of polyimide nanoparticles

**DOI:** 10.1098/rsos.231995

**Published:** 2024-10-02

**Authors:** Satoshi Kuretani, Kazushige Hori, Tomoyasu Hirai, Noboru Ohta, Kan Hatakeyama-Sato, Teruaki Hayakawa, Yuta Nabae

**Affiliations:** ^1^ Department of Materials Science and Engineering School of Materials and Chemical Technology, Tokyo Institute of Technology, 2-12-1-S8-36 Ookayama, Meguro-ku, Tokyo 152-8552, Japan; ^2^ Department of Applied Chemistry, Faculty of Engineering and Graduate School of Engineering, Osaka Institute of Technology, 5-16-1 Omiya, Asahi-ku, Osaka, 535-8585, Japan; ^3^ Japan Synchrotron Radiation Research Institute SPring-8,Hyogo, Sayo 679-5198, Japan

**Keywords:** polyimide, poly(amic acid), nanoparticle, precipitation, dispersant, SASX

## Abstract

Poly(amic ‍acid) ‍nanoparticles ‍prepared ‍by ‍‍precipitation ‍polymerization with a dispersant were evaluated by small-angle X-ray scattering (SAXS) and field-emission scanning electron microscopy (FE-SEM). The particle size evaluation of poly(amic acid) nanoparticles in the liquid phase by SAXS was performed to gain insight into the size control of poly(amic acid) nanoparticles, and showed good agreement with visual observation by FE-SEM, explaining the effect of the dispersant in obtaining polyimide nanoparticles with small particle size. This indicates that the particle size is maintained without change during the solvent evaporation process. The polyamide nanoparticles controlled by the dispersant effect maintained their size after imidization, and polyimide nanoparticles with a minimum radius of about 60 nm were prepared.

## 1. Introduction

Polymeric nanoparticles (NPs) with sizes ranging from one to hundreds of nanometers are of great interest in various fields, such as catalysis [1], electrical and optical devices [2,3] and drug carriers [4]. NPs are generally prepared using chain-growth polymerization, including radical, cationic and anionic polymerization [5,6]. However, such NPs suffer from a lack of thermal and chemical stability, which limits their industrial applications.

Polyimide is widely used as a super-engineering plastic because of its excellent thermal and chemical stability, mechanical strength and electrical insulation [7–10]. It is prepared via a polyaddition reaction and subsequent condensation. At present, little attention has been paid to the preparation of NPs via polyaddition. Poly(amic acid) NPs can be prepared using a polyaddition-based precipitation reaction and then converted into polyimide NPs by heat treatment [11–14]. We previously demonstrated that poly(amic acid) NPs with diameters of less than 50 nm can be obtained via precipitation polymerization in the presence of an amphiphilic dispersant [15,16]. However, the effect of dispersants on the size of the resultant NPs is incompletely understood.

Although poly(amic acid) NPs are prepared by precipitation polymerization in a liquid-phase matrix, the size and structure of the resulting NPs are generally evaluated by transmission electron microscopy (TEM) or scanning electron microscopy (SEM) in the dry state after the complete elimination of the polymerization solvent [17–25]. These microscopic techniques can clarify the structure of poly(amic acid) in the dry state, but its characteristics when dispersed in a liquid phase would provide more direct information on NP formation. Therefore, in this study, we performed small-angle X-ray scattering (SAXS) under synchrotron radiation on dispersions of poly(amic acid) in acetone immediately after precipitation polymerization and discussed the role of the dispersant in providing well-controlled NPs with a narrow size distribution.

## 2. Experimental

### 2.1. Materials

Pyromellitic dihydride (PMDA, >98.0%) and 4,4′–oxydianiline (ODA, >98.0%) were purchased from the Tokyo Chemical Industry and purified by sublimation before use. *N*,*N*-dimethyldodecylamine (DMDA) and acetone were purchased from Wako Pure Chemical Industry and used as-received, unless otherwise stated.

### 2.2. Fabrication of poly(amic acid) NPs

A solution of PMDA (0.2180 g, 1 mmol) and DMDA (as the dispersant) in acetone (50 ml) and a solution of ODA (0.2000 g, 1 mmol) in acetone (50 ml) were each stirred at 0°C for 20 min. The solution of ODA was added to the solution of PMDA and DMDA, and the mixture was further stirred at 0°C for 60 min. The concentration of the dispersant was varied in the range of 0–7.5 mmol l^−1^, and the resulting poly(amic acid) NPs were denoted PAA(*x*), where *x* represents the concentration of the dispersant (mmol l^−1^). The polymers without dispersant were also prepared in a similar manner and denoted as PAA(*x*) without dispersant.

### 2.3. Fabrication of polyimide NPs

Thermal imidization of PAA(*x*) was performed by drying the poly(amic acid) dispersion and then heat-treated at 240°C. The samples were denoted PI(*x*).

### 2.4. Small angle X-ray scattering

The PAA dispersions obtained immediately after precipitation polymerization were collected into quartz capillaries without any condensation or dilution and subjected to SAXS measurements under synchrotron radiation. The wavelength of the X-ray (*λ*) was set to 1.90 Å, and the scattering was detected using a PILATUS 2M detector (254 × 289 mm with a pixel size of 172 × 172 μm). The detector was placed 4188 mm away from the sample. The distance was calibrated with a silver behenate standard. The PAA particles were evaluated by subtracting the scattering intensity obtained by adding only acetone, which is the dispersion medium, to the quartz capillary from the scattering intensity of the PAA dispersion solution and the quartz capillary.

### 2.5. Measurements

Fourier transform infrared (FT-IR) spectroscopy was measured using a JASCO 4100 spectrometer using the KBr pellet method in the range of 400–4000 cm^−1^. FE-SEM was measured using a Hitachi S-5500 microscope at 1.0–2.0 kV. The inherent viscosity of PAA(*x*) was measured at a concentration of 0.5 g dl^−1^ with an Ostwald-type viscometer in *N,N*-dimethylacetamide (DMAc) at 30°C.

## 3. Results and discussion

**Scheme 1.**
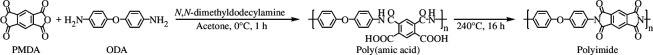
Synthetic route for obtaining poly(amic acid) and polyimide NPs.

Scheme 1 shows the synthetic route of poly(amic acid) and polyimide NPs. Poly(amic acid) was prepared via precipitation polymerization. An important criterion for successful precipitation polymerization is the use of a solvent that is good for the monomers but poor for the resulting polymers; therefore, acetone was selected as the solvent for this system. In the initial stage of polymerization, the PMDA + ODA + DMDA mixture was a homogeneous transparent solution, and a whitish precipitate was observed after tens of seconds. This mixture was stirred at 0°C for 60 min, and the dispersion was subjected to the required measurements.

**Figure 1.**
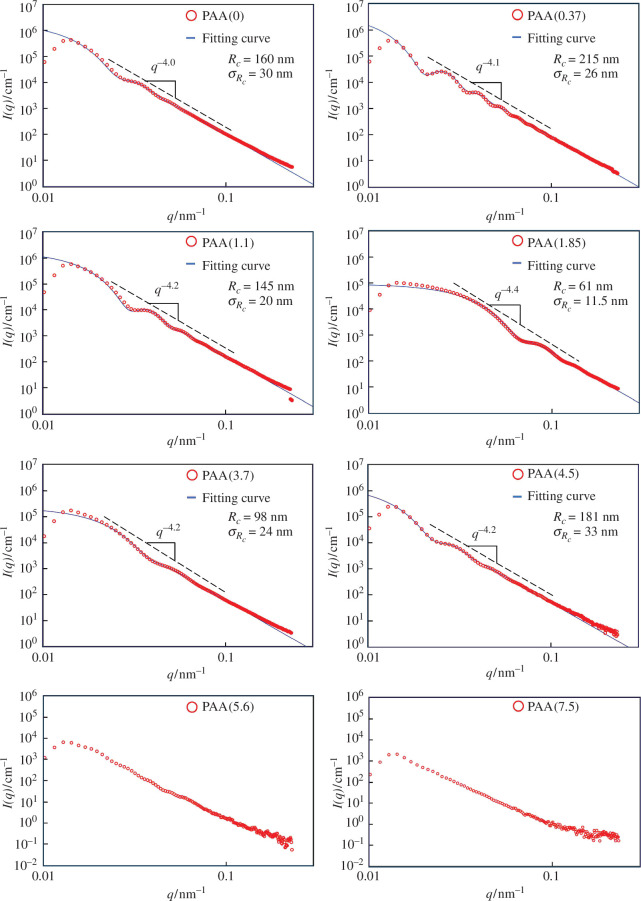
SAXS profiles of poly(amic acid) dispersions in acetone with various dispersant concentrations.


Figure 1 shows the SAXS profiles of the poly(amic acid) dispersions in acetone with various dispersant concentrations. The scattering vector ***q*** is defined as (4*π*/*λ*)sin*θ*. The majority of the SAXS profiles exhibited asymptotic behaviours with ***q***^−4^, which suggests the formation of spherical poly(amic acid) NPs [26]. The profiles of the spherical poly(amic acid)s were curve-fitted using equations (3.1–3.3), which have been proposed for isolated spheres [27,28].

(3.1) $$I\left(q\right)=k\int_{0}^{\infty }P\left(R\right){\left\{F_{\text{s}}\left(q\right)\right\}}^{2}\text{d}R,$$

(3.2) $$F_{\text{s}}\left(q\right)=\;V_{\text{s}}\left(\rho_{\text{s}}-\rho_{\text{m}}\right)\frac{3}{{\left(qR_{\text{c}}\right)}^{3}}\left\{\text{sin}\left(qR_{\text{c}}\right)-qR_{\text{s}}\text{cos}\left(qR_{\text{c}}\right)\right\},$$

(3.3) $$P\left(R\right)=\;\frac{1}{\sqrt{2\pi \sigma_{R_{\text{s}}}^{2}}}\text{exp}\left\{-\frac{{\left(R-R_{\text{s}}\right)}^{2}}{2\sigma_{R_{\text{s}}}^{2}}\right\}.$$

where *k* is the normalization constant, *V*_s_ is the volume of the sphere, *ρ*_s_ is the electron density of the sphere, *ρ*_m_ is the electron density of the medium, *R*_c_ is the radius of the sphere and *σ*_RS_ is the standard deviation of the sphere radius. Here, we used the values 435 and 261 e nm^−3^ for *ρ*_s_ and *ρ*_m_, respectively. Whether these profiles could be analysed using a core–shell model with a poly(amic acid) core and a dispersant shell has been raised [29,30], but doing so is difficult because the electron density of the alkyl chains in the dispersant was assumed to be very close to that of acetone (e.g. 261 e nm^−3^ for dodecane). The curve fittings (solid lines) of the NPs prepared with 0–4.5 mmol l^−1^ dispersant well reproduced the measured scattering profiles (open circles). The lower scattering intensity below ***q*** = 0.002 compared with the fitting curve is probably due to the beam stopper. Reasonable fittings for the PAA (5.6) and PAA (7.5) curves were difficult to obtain. The *R*_c_ and *σ*_RS_ values obtained from the best fitting for each profile are shown in figure 1.

**Figure 2.**
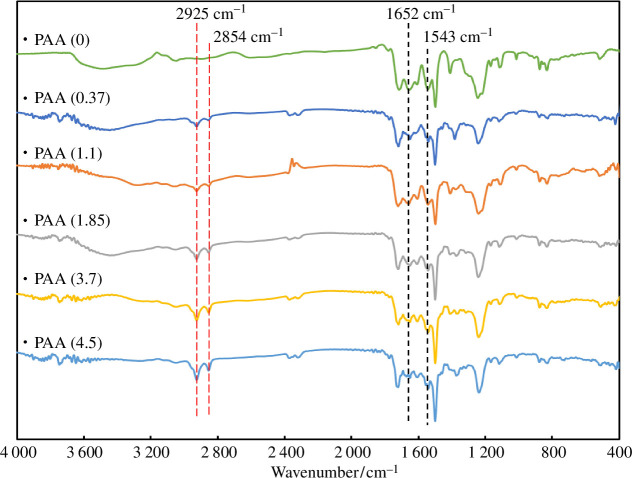
FT-IR spectra of poly(amic acid) NPs prepared with various dispersant concentrations.

To evaluate the dry state of poly(amic acid), the solution was cast onto a glass slide and subjected to FT-IR spectroscopy. The spectra shown in figure 2 suggest the formation of amide groups with absorption peaks at 1652 cm^−1^ (C=O stretching) and 1543 cm^−1^ (C–N–H stretching and bending). Moreover, the peaks at 2925 and 2854 cm^−1^ (C–H stretching) suggest the presence of the dispersant. The intensity of these signals increased with increasing dispersant concentration [31].

**Figure 3.**
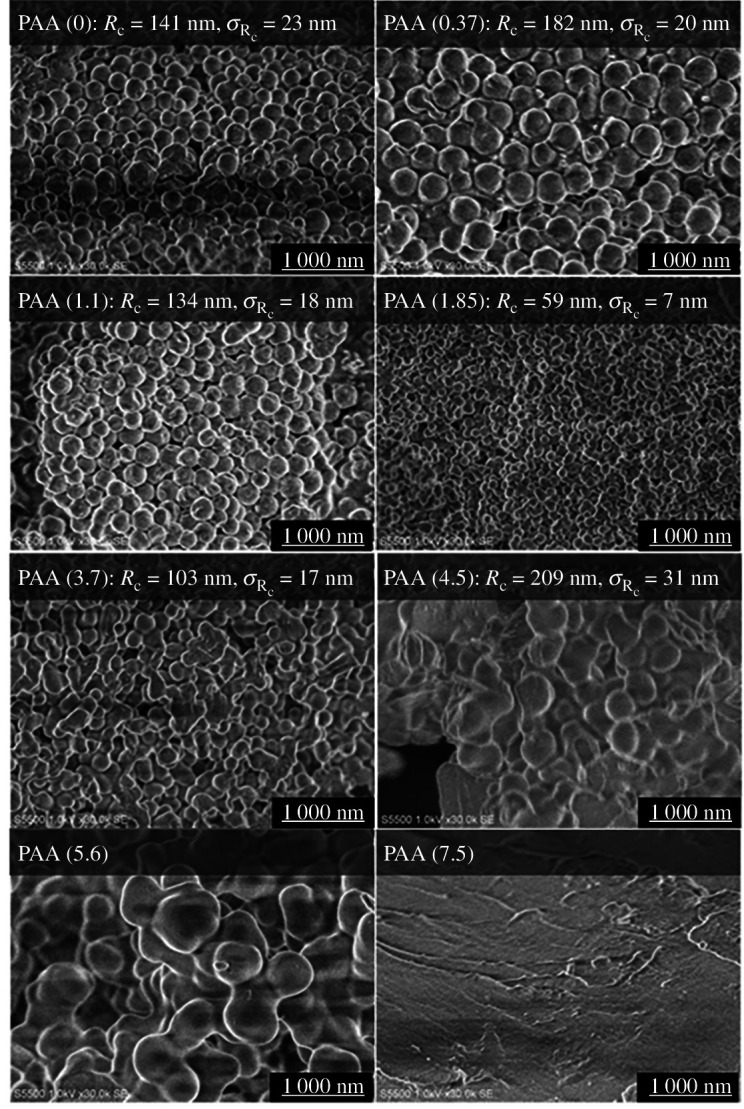
FE-SEM images of poly(amic acid) NPs prepared with various concentrations of dispersant.

Figure 3 shows FE-SEM images of the poly(amide acid) NPs prepared with various dispersant concentrations. The average diameters and standard deviations of the NPs were determined by manually counting hundreds of particles in the images. The samples prepared with less than 1.1 mmol l^−1^ dispersant showed many spherical NPs with radii of 130–180 nm. The smallest NPs with a radius of 59 nm and a narrow size distribution appeared at an adequate dispersant concentration. However, higher dispersant concentrations resulted in larger and non-uniform particles or nonspherical bulk poly(amic acid).

**Figure 4.**
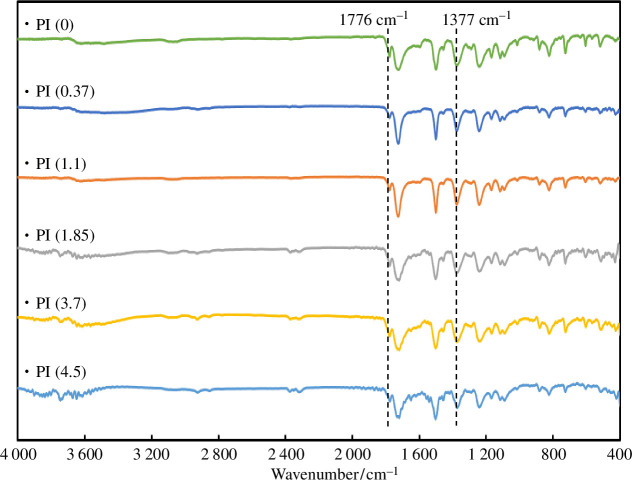
FT-IR spectra of polyimides prepared with various dispersant concentrations.

**Figure 5.**
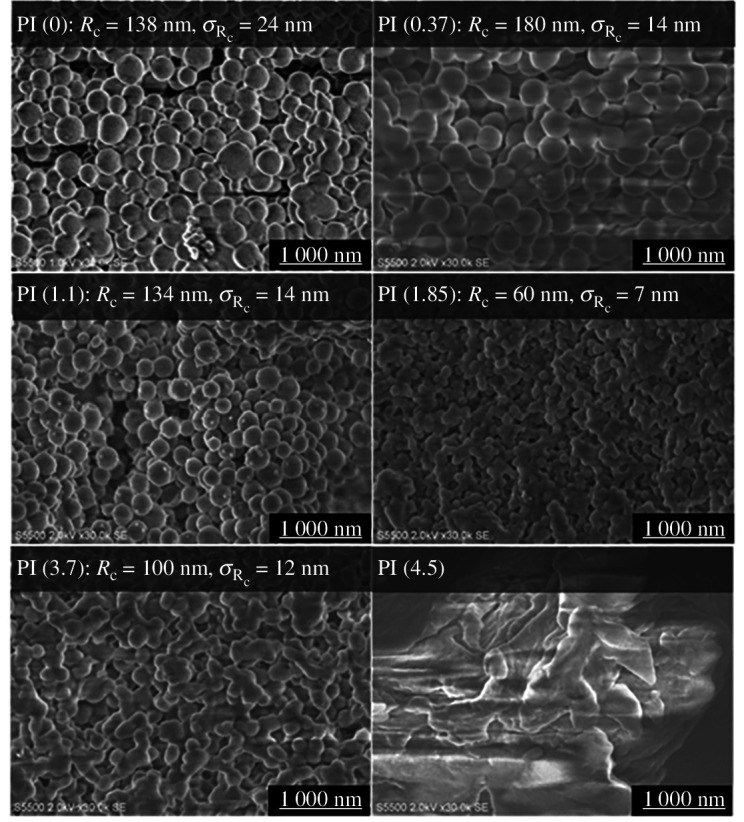
FE-SEM images of polyimides prepared with various dispersant concentrations.

To evaluate the dry state of polyimide, we evaporated the solvent in the poly(amic acid) dispersion and heat-treated the residue at 240°C for thermal imidization; here, the samples were denoted PI(*x*). Ring closure following imidization was confirmed by IR peaks at 1776 cm^−1^ (C=O stretching) and 1377 cm^−1^ (C-N-C stretching), as shown in figure 4. Notably, most of the signals derived from the dispersant disappeared after imidization. The FE-SEM images in figure 5 suggest that the particle size did not change during thermal imidization.

**Figure 6.**
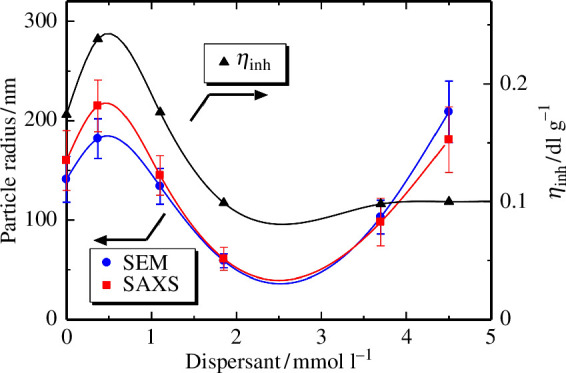
(Left) Particle radii evaluated by FE-SEM and SAXS, and (right) the inherent viscosity of poly(amic acid)s prepared with various dispersant concentrations.

As mentioned earlier, the morphology of poly(amic acid) NPs dispersed in a liquid matrix was investigated by SAXS, while those in the dry state were examined by SEM. The results are compared in figure 6. We anticipated that the morphologies in the dispersion and dry states would be quite different; however, interestingly, the results obtained in this study were in good agreement with each other. In addition, an optimal dispersant concentration for obtaining the smallest particle size with the narrowest size distribution was observed. The inherent viscosity was also investigated for the solutions of poly(amic acid)s prepared by dissolving the NPs into DMAc, to understand the effect of dispersant concentration on the molecular weight of the resulting poly(amic acid), and the results are plotted in figure 6. At concentrations below 2 mmol l^−1^, the particle size and inherent viscosity show a similar trend with the maximum values with PAA(0.37), suggesting the particle size and molecular weight of the poly(amic acid)s are strongly correlated. On the contrary, in the range of 2–4.5 mmol l^−1^ of dispersant, the particle sizes increased, whereas the inherent viscosity did not.

Considering the above experimental results, the formation of NPs during precipitation polymerization with various dispersant concentrations can be explained as follows. In the initial stage of polymerization, all monomer molecules are dissolved in the matrix solvent. Polymerization proceeds via sequential polymerization in the homogeneous solution to form oligomers and polymer chains. As the molecular weight of the oligomer/polymer increases, the system reaches a solubility limit at a certain point and polymer precipitation occurs. The precipitates then tend to form uniform spherical particles that minimize the surface energy of the particle/matrix interface. A small concentration of dispersant (<0.5 mmol l^−1^) probably works as a solubilizer for the polymer chains and results in larger particle sizes of NPs with longer chains compared with the NPs without the dispersant. On the contrary, in the presence of an adequate dispersant concentration, the particle/matrix interface is stabilized by the dimethylamino groups interacting with the polar groups of poly(amic acid) and the alkyl chains interacting with the matrix solvent. This stabilization effect results in the precipitation of particles at an early stage of polymerization and enhances the formation of smaller NPs. Once such precipitation occurs, the size of the particles does not increase because they are isolated by the stabilizer, and the morphology of the particles is retained throughout subsequent polymerization and solvent evaporation. Finally, thermal imidization immobilizes the morphology of the NPs such that they do not collapse even after dispersant elimination. By contrast, in the presence of excess dispersant, the precipitation starts in a similar manner to that with lower concentrations of dispersant, but the isolation effect of the stabilizer does not work well, leading to the formation of non-uniform particles or nonspherical precipitates.

## 4. Conclusions

Poly(amic acid) was dispersed in a liquid matrix and the resulting poly(amic acid) NPs were investigated by synchrotron SAXS and SEM. The experiments revealed that the NP morphology in the dispersion state agreed well with that in the dry state. This finding suggests that the morphology formed at an early stage of polymerization is retained throughout polymerization and subsequent solvent evaporation. The particle size of the NPs can be controlled by employing a suitable dispersant concentration, which contributes to the stabilization of the particle/matrix interface. Excess dispersant is not beneficial to precipitation polymerization because the stabilization effect does not work well. Furthermore, studies will be performed to fabricate smaller polyimide NPs and expand the applications of such NPs in various industrial fields.

## Data Availability

The source data for all figures will be provided as electronic supplementary material [32].
